# Supplementary material for: Identifying determinants of under-5 mortality in Bangladesh: A machine learning approach with BDHS 2022 data
Source: PLoS One. 2025 Jun 11;20(6):e0324825. doi: 10.1371/journal.pone.0324825 (PMC12157215; doi:10.1371/journal.pone.0324825)
Supplement: Attachment — Submitted filename: point by point Response - Review 2.pdf [file pone.0324825.s003.pdf]

Response to Editors & Reviewers

"Identifying Determinants of Under-5 Mortality in Bangladesh: A Machine Learning Approach with BDHS 2022 Data"

Ref: Manuscript Number: [PONE-D-24-48508R1] - [EMID:c5fe75877697e9f4]

Dear Editors,

Regarding our manuscript, we thank you for the comments sent on 11 March 2025. My co-authors and I have revised the manuscript accordingly and would like it to be reconsidered for publication. As requested, we have included point-by-point responses to editors' and reviewers' comments below.

Please let us know if anything further is required at this time, and we thank you very much for considering our revised manuscript.

Shayla Naznin

**Editors comments**	**Response**
Thank you for submitting your manuscript to PLOS ONE. After careful consideration, we feel that it has merit but does not fully meet PLOS ONE's publication criteria as it currently stands. Therefore, we invite you to submit a revised version of the manuscript that addresses the points raised during the review process.	Thank you very much for your feedback! We have endeavored to address all of the reviewers' comments as detailed below to further strengthen the paper.
**Reviewer 1**	
The authors have revised the manuscript in response to my initial review, and all of my suggestions have been adequately addressed. It is of my opinion that the manuscript is suitable for publication.	Thank you for this careful reading and positive feedback. It will help us a lot.
**Reviewer 2**	
• Title and Abstract: The title is clear, and the abstract effectively summarizes the study's purpose, methodology, and significant findings. However, a more explicit statement about the implications of the findings could enhance clarity.	Thank you for this careful reading this has been revised kindly address the line #32-37

• Introduction: The introduction provides sufficient background on under-5 mortality and articulates the research objectives clearly. However, the significance of machine learning in this context could be emphasized more.	Thank you for this careful reading this has been revised kindly address the line #63-93
• Methods: The methods are described in detail, allowing for replication. Various machine learning models are explored; this comprehensive approach is appropriate for addressing the research question. A more detailed rationale for the choice of metrics for evaluation could enhance the methods section.	Thank you for this careful reading this has been revised kindly address the line #247-278
o Study Design: The study employs an appropriate design, leveraging machine learning models suited to the data type.	Thank you for this careful reading and positive feedback. It will help us a lot.
o Data Analysis: The statistical methods are appropriate and correctly applied. Reproducibility: The details provided allow for reproducibility	Thank you for this careful reading and positive feedback. It will help us a lot.
• Ethics: The stated compliance with ethical standards, including data source legality, is appropriate.	Thank you for this careful reading and positive feedback. It will help us a lot.
• Results: The results are presented clearly and supported by relevant data. Figures and tables are well-designed, aiding comprehension. Including confidence intervals alongside performance metrics could provide more context and robustness.	Thank you for this careful reading this has been revised kindly address the line #365-378
• Discussion: The discussion effectively interprets results within existing literature and acknowledges limitations, such as the cross-sectional nature of the data. A more detailed comparison with similar studies would strengthen the discussion.	Thank you for this careful reading this has been revised kindly address the line #439-528
• Conclusion: The conclusions drawn are supported by the data and effectively address the research objectives, reiterating key findings.	Thank you for this careful reading and positive feedback. It will help us a lot.
